# Using Genetic Variants to Evaluate the Causal Effect of Plasma Phospholipid Fatty Acids on Breast Cancer and Prostate Cancer: A Mendelian Randomization Study

**DOI:** 10.3389/fgene.2021.664498

**Published:** 2021-06-30

**Authors:** Ze Yang, Jingjia Li, Yandi Sun, Zihao Qu, Yindan Lin, Lihong Zhang, Qian He, Xueyao Jia, Mashaal Ahmad, Xueyun Zhang, Yan Luo

**Affiliations:** ^1^Department of Biochemistry and Cancer Institute of the Second Affiliated Hospital, Zhejiang University School of Medicine, Hangzhou, China; ^2^Key Laboratory of Cancer Prevention and Intervention of China National Ministry of Education, Hangzhou, China; ^3^Department of Orthopedic Surgery, The Second Affiliated Hospital, Zhejiang University School of Medicine, Hangzhou, China; ^4^Orthopedic Research Institute of Zhejiang University, Hangzhou, China

**Keywords:** risk factors, Mendelian randomization, prostate cancer, breast cancer, plasma phospholipid fatty acids

## Abstract

**Background:**

Observational studies indicate that phospholipid fatty acids (FAs) have an impact on the etiology in cancers, but the results are conflicting. We aimed to investigate the causal association of phospholipid FAs with breast cancer and prostate cancer.

**Methods:**

Fourteen single nucleotide polymorphisms (SNPs) were selected as instrumental variables to predict the level of 10 phospholipid FAs from Genome-wide association studies (GWAS). We obtained the summary statistics for the latest and largest GWAS datasets for breast cancer (113,789 controls and 133,384 cases) and prostate cancer (61,106 controls and 79,148 cases) from the Breast Cancer Association Consortium (BCAC) and Prostate Cancer Association Group to Investigate Cancer Associated Alterations in the Genome (PRACTICAL) consortium. Two-sample Mendelian randomization analysis was applied.

**Results:**

The results demonstrate that the 10 individual plasma phospholipid FAs are not significantly associated with breast cancer risk and prostate cancer risk.

**Conclusion:**

The evidence is insufficient to support the causal association of the 10 individual plasma phospholipid FAs with breast cancer and prostate cancer.

## Introduction

Fatty acids (FAs) are involved in various physiological processes, including maintaining cell membrane stability, forming raft lipid to regulate signal transduction, the process of inflammation, and even hormone synthesis (Lands, 2014; Fritsche, 2015; Calder, 2020). The two hormone-related cancers, breast cancer and prostate cancer, are the leading causes of death and the most common cancers among women and men (Bray et al., 2018; Siegel et al., 2019). As FAs are modifiable factors that strongly relate to dietary intake, whether FAs play a role in tumorigenesis has been a focus of investigation. However, overall findings from the epidemiological studies are conflicting.

Epidemiological studies suggested an inverse association of ω-3 polyunsaturated fatty acids (PUFA) (Zheng et al., 2013; Hirko et al., 2018; Yang et al., 2019), a positive association of ω-6 PUFA (Fabian et al., 2015; Kiyabu et al., 2015), and saturated fatty acids (SFA) with breast cancer risk (Brennan et al., 2015; Xia et al., 2015; Hirko et al., 2018). However, ω-3 PUFA is shown to be a risk factor for prostate cancer, whereas ω-6 PUFA and SFA are the protective factors by several observational studies (Dahm et al., 2012; Brasky et al., 2013; Crowe et al., 2014). Furthermore, several studies indicate that both the above FAs and monounsaturated fatty acids (MUFA) are not associated with breast cancer risk (Sanders, 2014; Cao et al., 2016; Zhou et al., 2016). These contradictory results are largely based on observational studies that are prone to reverse causality and residual confounding. A recent large randomized control trial (RCT, *n* = 25,871) with follow-up for an average of 5.3 years concluded that ω-3 PUFA supplementation did not reduce major cardiovascular events and overall cancer incidence (Manson et al., 2019), while the specific data of large RCT investigating the effects of PUFA, MUFA, and SFA on the two hormone-related cancer is limited.

To overcome the aforementioned problems, Mendelian randomization (MR) analysis is widely applied to investigate the causality between the exposure and outcome. MR analysis uses single nucleotide polymorphisms (SNPs) for predicting the level of exposure to determine its causal effects on the outcome (Emdin et al., 2017; Gala and Tomlinson, 2020). According to the Mendel’s genetic law, SNPs are randomly assigned during gamete formation. The MR approach, therefore, can be thought of as a “natural” RCT (Gala and Tomlinson, 2020). Herein, we used the MR approach to investigate the causal association of 10 individual circulating phospholipid FAs with breast cancer and prostate cancer, including α-linolenic acid (ALA), docosahexaenoic acid (DHA), oleic acid (OA), palmitic acid (PA), eicosapentaenoic acid (EPA), docosapentaenoic acid (DPA), linoleic, acid (LA), arachidonic acid (AA), palmitoleic acid (POA), and stearic acid (SA).

## Materials and Methods

### Selection of SNPs

**FIGURE 1:**
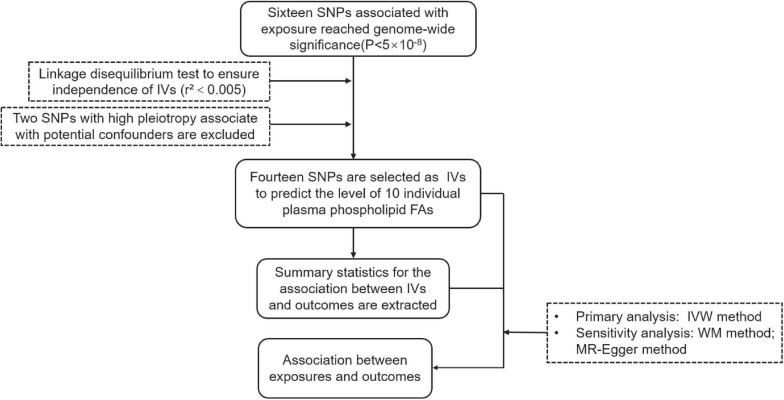
The flow chart of this study. IV, Instrumental variable; IVW, Inverse-variance-weighted; SNP, Single nucleotide polymorphism; WM, Weighted Median.

The instrumental variables (IVs) were built on the largest genomewide association studies (GWAS) on circulating phospholipid FAs from the Cohorts for Heart and Aging Research in Genomic Epidemiology consortium, in which the participants are of European origin (Lemaitre et al., 2011; Wu et al., 2013; Guan et al., 2014). The IVs are associated with 10 individual plasma phospholipid FAs mentioned above. SNPs at the genomewide significance (*P* < 5 × 10^–8^) of the association with the phospholipid FAs were subsequently searched on the PhenoScanner V2 website to determine whether the SNPs were related to the potential confounders between plasma phospholipid FAs and the two types of cancer. Furthermore, a primary assumption in MR analysis is that the IVs influence the outcome only *via* the exposure, while other pathways involved between the IVs and outcome can cause a biased result. Given the above, the two SNPs with high pleiotropy are associated with body mass index and alcohol consumption were excluded (rs780093 and rs780094). To ensure the selected SNPs were independently (*r*^2^<0.005) associated with the corresponding FAs, we performed a linkage disequilibrium test on the LD-link website (population: CEU)^1^ (Supplementary Tables 1, 2). Finally, 14 distinct SNPs were selected as the previous studies (Yuan et al., 2019; Yuan and Larsson, 2020) to predict the serum phospholipid level of ω-3 PUFAs (*n* = 8,866) (Lemaitre et al., 2011), ω-6 PUFAs (*n* = 8,631) (Guan et al., 2014), and MUFA and SFAs (*n* = 8,961) (Wu et al., 2013). The characteristics of the IVs are shown in Table 1, and the brief overview of the MR study design is shown in Figure 1.

**TABLE 1:** Characteristics of the instrumental variables (IVs) associated with plasma phospholipid fatty acids (FAs) and the associations with breast cancer and prostate cancer.

**Category of FA**	**FA**		**IV**	**Chr**	**Nearby gene**	**EA**	**Association with FAs**			**Association with breast cancer**			**Association with prostate cancer**		
							**Beta**	**SE**	***P*-value**	**Beta**	**SE**	***P*-value**	**Beta**	**SE**	***P*-value**
ω-3 PUFA	ALA	18:3(ω-3)	rs174547	11	FADS1	C	0.02	0.001	3.5E-64	–0.0082	0.0064	0.1954	–0.0029	0.0085	0.7288
	EPA	20:5(ω-3)	rs3798713	6	ELOVL2	C	0.04	0.005	1.9E-12	–0.0013	0.0060	0.8352	0.0101	0.0081	0.2122
			rs174538	11	FADS1/C11orf10	G	0.08	0.005	5.4E-58	0.0074	0.0065	0.2548	0.0063	0.0087	0.4674
	DPA	22:5(ω-3)	rs3734398	6	ELOVL2	C	0.04	0.003	9.7E-43	–0.0012	0.0060	0.8466	0.0122	0.0081	0.1304
			rs174547	11	FADS1	T	0.08	0.003	3.8E-154	0.0082	0.0064	0.1954	0.0029	0.0085	0.7288
	DHA	22:6(ω-3)	rs2236212	6	ELOVL2	G	0.11	0.014	1.3E-15	0.0011	0.0061	0.8602	–0.0126	0.0081	0.1198
ω-6 PUFA	LA	18:2(ω-6)	rs10740118	10	JMJD1C	G	0.25	0.05	8.1E-09	0.0182	0.0061	0.0030	0.0031	0.0081	0.7006
			rs174547	11	FADS1	C	1.47	0.05	5E-274	–0.0082	0.0064	0.1954	–0.0029	0.0085	0.7288
			rs16966952	16	NTAN1	G	0.35	0.04	1.2E-15	–0.0080	0.0071	0.2592	0.0186	0.0088	0.03536
	AA	20:4(ω-6)	rs174547	11	FADS1	T	1.69	0.02	3.3E-971	0.0082	0.0064	0.1954	0.0029	0.0085	0.7288
			rs16966952	16	NTAN1	G	0.2	0.03	2.4E-10	–0.0080	0.0071	0.2592	0.0186	0.0088	0.03536
ω-7 MUFA	POA	16:1(ω-7)	rs6722456	2	RN7SKP93	G	0.05	0.009	4.1E-08	0.0297	0.0206	0.1499	–0.0079	0.0289	0.7845
			rs603424	10	SCD/PKD2L1	G	0.03	0.004	5.7E-15	–0.0071	0.0077	0.3621	–0.0082	0.0106	0.4389
			rs11190604	10	HIF1AN	G	0.02	0.004	5.7E-09	0.0010	0.0073	0.8878	0.001	0.0098	0.9185
			rs102275	11	FADS1/2	C	0.02	0.003	6.6E-13	–0.0081	0.0063	0.2009	–0.0007	0.0084	0.9304
ω-9 MUFA	OA	18:1(ω-9)	rs102275	11	FADS1/2	C	0.23	0.02	2.2E-32	–0.0081	0.0063	0.2009	–0.0007	0.0084	0.9304
SFA	PA	16:0	rs2391388	1	ALG14	C	0.18	0.03	2.7E-11	0.0060	0.0062	0.3291	0.0025	0.0083	0.7611
	SA	18:0	rs6675668	1	ALG14	G	0.17	0.02	2.2E-18	–0.0045	0.0062	0.4607	–0.002	0.0082	0.8058
			rs11119805	1	LPGAT1	T	0.17	0.03	2.8E-09	0.0028	0.0094	0.7687	0.0107	0.0126	0.397
			rs102275	11	FADS1/2	T	0.18	0.02	1.3E-20	0.0081	0.0063	0.2009	0.0007	0.0084	0.9304

**Chr, chromosome; EA, effect allele; Beta, per allele effect on exposure or outcomes; SE, standard error; PUFA, polyunsaturated fatty acid; MUFA, monounsaturated fatty acid; SFA, saturated fatty acid; ALA α-linolenic acid;, EPA, eicosapentaenoic acid; DPA, docosapentaenoic acid; DHA, docosahexaenoic acid; LA, linoleic acid; AA, arachidonic acid; POA, palmitoleic acid; OA, oleic acid; PA, palmitic acid; SA, stearic acid.**

### Genetic Association With Outcomes

Summary statistics for breast cancer were obtained from the largest and latest GWAS meta-analysis from The Breast Cancer Association Consortium comprising of 133,384 cases and 113,789 controls^2^ (Zhang et al., 2020). The participants were limited to women of European ancestry. The summary data for prostate cancer was derived from the hitherto largest meta-GWAS with 79,148 cases and 61,106 controls of European ancestry from Prostate Cancer Association Group to Investigate Cancer-Associated Alterations in the Genome consortium^3^ (Schumacher et al., 2018). The summary information of GWAS on outcomes are displayed in Supplementary Table 3. The genetic association between plasma phospholipid FA and the outcomes are shown in Table 1.

The participants have provided written consent, and all the studies contributing data to our MR analysis were approved by the relevant ethical review boards.

### Statistical Analysis

The primary MR analysis of the causal association between plasma phospholipid FAs and cancers was performed by inverse-variance-weighted (IVW) approach to estimate each IV’s combined causal effects. The IVW approach evaluates the combined effect by calculating the Wald ratio of each SNPs and then using the corresponding inverse variance as weights for meta-analysis (Lee et al., 2016). There is only one SNP as IV for ALA, DHA, OA, and PA; therefore, the Wald ratio was directly used to calculate effect in the IVW analysis. By calculating the median value of the IVs’ estimates, a weighted median (WM) analysis was used in sensitivity analysis (Bowden et al., 2016). Furthermore, we test the pleiotropic effects through the MR-Egger approach, which examines whether the intercept of the association between plasma phospholipids and cancers differs from zero (Bowden et al., 2015).

All statistical analyses were conducted by R version 4.0.2 and R package “MendelianRandomization.”

## Results

### Plasma Phospholipid FAs and Breast Cancer

**FIGURE 2:**
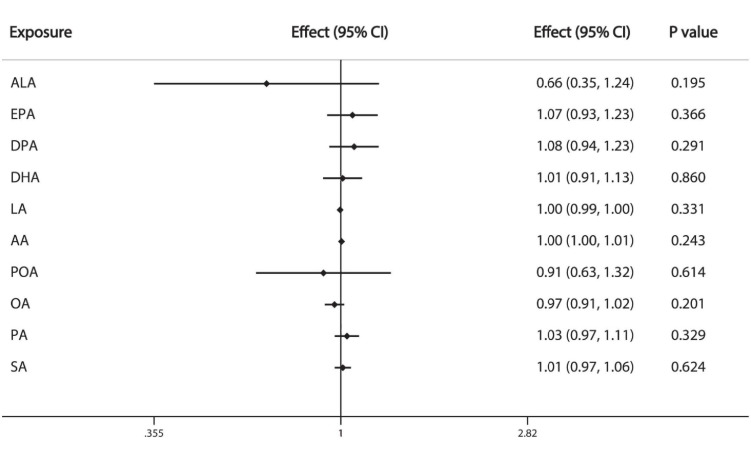
Causal associations between plasma phospholipid FAs and breast cancer risk. CI: confidence interval; Effect: Odds ratio.

We used IVW method as primary analysis, the results of which show that the 10 individual FAs examined are not significantly associated with breast cancer: ALA (OR = 0.66, 95%CI: 0.35, 1.24), EPA (OR = 1.07, 95%CI: 0.93, 1.23), DPA (OR = 1.08, 95%CI: 0.94, 1.23), DHA (OR = 1.01, 95%CI: 0.91, 1.13), LA (OR = 1.00, 95%CI: 0.99, 1.00), AA (OR = 1.00, 95%CI:1.00, 1.01), POA (OR = 0.91, 95%CI: 0.63, 1.32), OA (OR = 0.97, 95%CI: 0.91, 1.02), PA (OR = 1.03, 95%CI: 0.97, 1.11), SA (OR = 1.01, 95%CI: 0.97, 1.06) (Figure 2).

### Plasma Phospholipid FAs and Prostate Cancer

**FIGURE 3:**
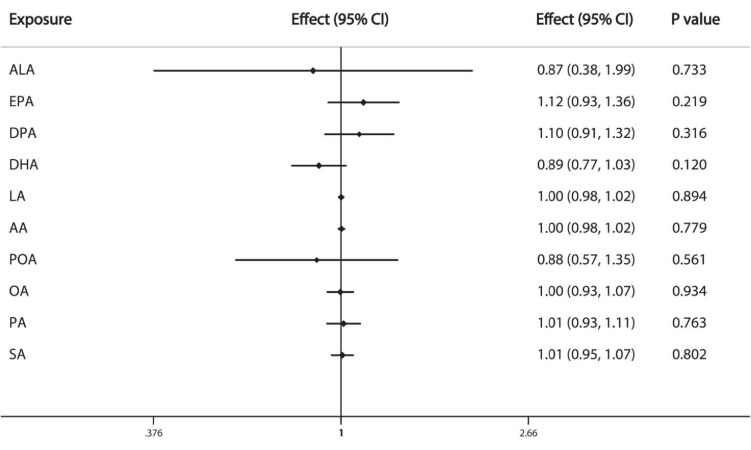
Causal associations between plasma phospholipid FAs and prostate cancer risk. CI: confidence interval; Effect: Odds ratio.

The results of IVW analysis demonstrate that the FAs are not causally associated with prostate cancer: ALA (OR = 0.87, 95%CI: 0.38, 1.99), EPA (OR = 1.12, 95%CI: 0.93, 1.36), DPA (OR = 1.10, 95%CI: 0.91, 1.32), DHA (OR = 0.89, 95%CI: 0.77, 1.03), LA (OR = 1.00, 95%CI: 0.98, 1.02), AA (OR = 1.00, 95%CI:0.98, 1.02), POA (OR = 0.88, 95%CI: 0.57, 1.35), OA (OR = 1.00, 95%CI: 0.93, 1.07), PA (OR = 1.01, 95%CI: 0.93, 1.11), SA (OR = 1.01, 95%CI: 0.95, 1.07) (Figure 3).

### Sensitivity Analysis

WM and MR-Egger analysis could not be performed to test the pleiotropy of EPA, DPA, DHA, LA, AA, OA, and PA because the IVs were less than three SNPs. The sensitivity analysis was conducted only to LA, POA, and SA; the estimated effects of IVW analyses were similar to the results calculated by WM, MR-Egger, and MR-Egger intercept methods (Supplementary Tables 4, 5). The intercept values of the MR-Egger analysis did not significantly differ from zero. The sensitivity analysis suggested that the pleiotropy did not bias our results.

## Discussion

We used the MR approach to investigate the causal association of the 10 individual plasma phospholipid FAs with breast cancer and prostate cancer, including MUFA, SFA, ω-3, and ω-6 PUFA. Our MR analysis indicates the 10 individual FAs we examined are not significantly associated with the breast cancer risk and prostate cancer risk.

Among the different types of FAs, ω-3 PUFA is the most controversial and has been considered as a protective factor for breast cancer (Zheng et al., 2013; Fabian et al., 2015; Hirko et al., 2018; Yang et al., 2019). Higher dietary intake of ω-3 PUFA has been shown to negatively associated with the breast cancer risk in meta-analyses (Zheng et al., 2013; Yang et al., 2019). Given the complexity of digestion, absorption and conversion of the dietary FAs, the level of circulating FAs and dietary intake of FAs are not completely equal. Circulating FAs constitute the endogenous exposure of bioavailable FA to individuals (Jenab et al., 2009). In a large case-control study (N_*case*_ = N_*control*_ = 2,982) nested within the European Prospective Investigation into Cancer and Nutrition study, the researchers found no significant association between plasma phospholipid ω-3 PUFA between breast cancer risk (Chajes et al., 2017). Another nested case-control (N_*case*_ = N_*control*_ = 794) study in Nurses’ Health Study II demonstrated that erythrocyte membrane FAs, which reflected dietary intake for several months were not associated with breast cancer overall, while the subgroup analysis suggested a protective effect of DPA and EPA but not DHA among overweight women (Hirko et al., 2018). A more recently published meta-analysis indicates circulating ω-3 PUFA is significant associated with a lower breast cancer risk (Yang et al., 2019). However, the large RCT in New England of Medicine in 2019, Manson et al. reported that ω-3 PUFA supplementation (460 mg of EPA and 380 mg of DHA per day) did not reduce the overall cancer risk (HR:1.03, 95%CI:0.93-1.13), as well as the breast cancer risk (HR:0.90, 95%CI:0.70-1.16) and prostate cancer risk (HR:1.15, 95%CI:0.94-1.39) (Manson et al., 2019). Our MR analysis also demonstrates that ω-3 PUFA is not causally associated with breast cancer and prostate cancer (Figures 2, 3).

Whether FAs play a role in prostate carcinogenesis is similarly controversial. Meta-analyses have attempted to clarify the association between FAs and prostate cancer risk; a meta-analysis demonstrates that dietary intake of ALA reduces the risk (Fu et al., 2015), while several meta-analyses indicate that dietary intake of PUFA or SFA is not associated with prostate cancer incidence (Szymanski et al., 2010; Alexander et al., 2015; Aucoin et al., 2017). Given that the interpretation on the results of meta-analyses was complicated by interstudy heterogeneity, Wu et al. recently reported the data from a 24-year prospective cohort study (*n* = 47,885), which suggests that higher intake of ALA is not associated with the lethal prostate cancer risk after the introduction of PSA screening (Wu et al., 2018). However, the findings of studies on circulating FAs are contrary to the expected anti-inflammatory effect of ω-3 PUFA. Observational studies suggest that total ω-3 PUFA or individual ω-3 PUFA (i.e., ALA, DPA, EPA, DHA) are risk factors for prostate cancer (Crowe et al., 2008, 2014; Simon et al., 2009; Brasky et al., 2011, 2013; Dahm et al., 2012; Fu et al., 2015; Huang et al., 2019), whereas stearic acid (Crowe et al., 2008, 2014) and ω-6 PUFA (Brasky et al., 2013) are marginally inversely associated with prostate cancer. Relatively recent meta-analysis results do not support an association between serum ω-3 PUFA with prostate cancer risk (Alexander et al., 2015). Our MR analysis results demonstrate that plasma phospholipid FAs, including ω-3 and ω-6 PUFA, SFA, and MUFA, may not have a role in prostate cancer etiology.

Researchers have applied the MR approach to investigate the causal association between plasma phospholipid FAs and several diseases, including atrial fibrillation (Yuan and Larsson, 2019), orthopedic diseases (Yuan et al., 2020), cardiovascular diseases (Yuan et al., 2019), and type 2 diabetes (Yuan and Larsson, 2020). To our knowledge, we first used MR analysis to address this highly controversial issue, which avoids the reverse causality and confounders that result in bias in observational studies. Previous cohort studies assessing the association of FAs with tumorigenesis primarily used dietary questionnaires to estimate specific FA intake or directly detected the level of FAs through a single collection of blood samples several years ago. Instead, we used GWAS datasets with a large sample size and measured the genetic predisposing level of FAs by SNPs that reflects a long-term bioavailable exposure, which enabled a relatively reliable result. However, a major limitation of our study is that some IVs (rs174547, rs16966952, rs102275) are related to more than one individual plasma phospholipid FAs, which leads to a lower probability to unravel the association between specific FAs and cancers. Another limitation is that seven exposures (DPA, DHA, EPA, ALA, AA, OA, and PA) were excluded in the sensitivity analysis because the IVs associated with them is less than three, which did not reach the requirement of MR-Egger and WM analysis. Given that we performed the MR analysis using GWAS datasets of European descendants, the conclusion we made may not be applicable to other populations.

In conclusion, our MR analysis indicates that little evidence supports the causal association of the 10 plasma phospholipid FAs with breast cancer and prostate cancer.

## Data Availability Statement

The original contributions presented in the study are included in the article/Supplementary Material, further inquiries can be directed to the corresponding author/s.

## Ethics Statement

Ethical review and approval was not required for the study on human participants in accordance with the local legislation and institutional requirements. Written informed consent for participation was not required for this study in accordance with the national legislation and the institutional requirements.

## Author Contributions

ZY, JL, YS, YLu, and XZ conceived the study design and drafted the manuscript. ZY, JL, YS, and ZQ participated in data extraction and data analysis. YLi, LZ, QH, XJ, and MA did the data checking and analysis. All authors critically reviewed the manuscript.

## Conflict of Interest

The authors declare that the research was conducted in the absence of any commercial or financial relationships that could be construed as a potential conflict of interest.

## Footnotes

1 https://ldlink.nci.nih.gov/

2 http://bcac.ccge.medschl.cam.ac.uk/

3 http://practical.icr.ac.uk/blog/

## Abbreviations

AA: Arachidonic acid
ALA: α -linolenic acid
CI: Confidence interval
DHA: Docosahexaenoic acid
DPA: Docosapentaenoic acid
EPA: Eicosapentaenoic acid
FA: Fatty acid
GWAS: Genome-wide association studies
IV: Instrumental variable
IVW: Inverse-variance-weighted
LA: linoleic acid
MR: Mendelian randomization
MUFA: Monounsaturated fatty acid
OR: Odds ratio
OA: Oleic acid
PA: Palmitic acid
PSA: Prostate-specific antigen
POA: Palmitoleic acid
PUFA: Polyunsaturated fatty acid
RCT: Randomized controlled trial
SA: Stearic acid
SFA: Saturated fatty acid
SNP: Single nucleotide polymorphism
WM: Weighted median.
