# Supplementary material for: Using Genetic Variants to Evaluate the Causal Effect of Plasma Phospholipid Fatty Acids on Breast Cancer and Prostate Cancer: A Mendelian Randomization Study
Source: Front Genet. 2021 Jun 30;12:664498. doi: 10.3389/fgene.2021.664498 (PMC8278063; doi:10.3389/fgene.2021.664498)
Supplement: Supplementary file 1 [file Data_Sheet_1.docx]

**Table S1 Calculation of linkage disequilibrium of the selected SNP on chromosome 1**

| SNP | rs6675668 | rs11119805 |
| --- | --- | --- |
| rs6675668 | 1.0 | 0.002 |
| rs11119805 | 0.002 | 1.0 |

The estimates of linkage disequilibrium are reported as r^2^ values in the table.

Population = (CEU) Utah Residents from North and West Europe; r^2^ < 0.005.

**Table S2 Calculation of linkage disequilibrium of the selected SNP on chromosome 10**

| SNP | rs603424 | rs11190604 |
| --- | --- | --- |
| rs603424 | 1.0 | 0.002 |
| rs11190604 | 0.002 | 1.0 |

The estimates of linkage disequilibrium are reported as r^2^ values in the table.

Population = (CEU) Utah Residents from North and West Europe; r^2^ < 0.005.

**Table S3 The characteristics of GWAS studies on the included outcomes**

| Outcome | Consortium | Total population | Cases/controls | Ethnicity | References |
| --- | --- | --- | --- | --- | --- |
| Breast cancer | BCAC | 247173 | 133384/113789 | European | Genome-wide association study identifies  32 novel breast cancer susceptibility loci from overall and subtype-specific analyses. [28] |
| Prostate cancer | PRATICAL | 140254 | 79148/ 61106 | European | Association analyses of more than 140,000 men identify 63 new prostate cancer susceptibility loci. [29] |

BCAC, Breast Cancer Association Consortium; PRATICAL, Prostate Cancer Association Group to Investigate Cancer-Associated Alterations in the Genome.

**Table S4 Weighted median and MR-Egger analysis for genetic associations between exposures and breast cancer risk**

| Method | Weighted median | MR-Egger | |
| --- | --- | --- | --- |
|  |  | Estimate | Intercept |
| LA | | | |
| Estimate (95% CI) | -0.006 (-0.014, 0.002) | -0.014 (-0.049, 0.020) | 0.012 (-0.019, 0.043) |
| P value | 0.170 | 0.421 | 0.460 |
| POA | | | |
| Estimate (95% CI) | -0.191 (-0.592, 0.209) | 0.617 (-0.700, 1.934) | -0.018 (-0.051, 0.014) |
| P value | 0.349 | 0.358 | 0.270 |
| SA | | | |
| Estimate (95% CI) | 0.01 (-0.038, 0.073) | 1.042 (-0.554, 2.638) | -0.179 (-0.457, 0.098) |
| P value | 0.538 | 0.201 | 0.206 |

**Table S5 Weighted median and MR-Egger analysis for genetic associations between exposures and prostate cancer risk**

| Method | Weighted median | MR-Egger | |
| --- | --- | --- | --- |
|  |  | Estimate | Intercept |
| LA | | | |
| Estimate (95% CI) | -0.001(-0.012, 0.010) | -0.01(-0.034, 0.014) | 0.013(-0.008, 0.034) |
| P value | 0.889 | 0.401 | 0.230 |
| POA | | | |
| Estimate (95% CI) | -0.132(-0.637, 0.373) | -0.473(-2.064, 1.118) | 0.009(-0.030, 0.048) |
| P value | 0.608 | 0.560 | 0.658 |
| SA | | | |
| Estimate (95% CI) | 0.0004(-0.068, 0.067) | -0.108(-2.235, 2.019) | 0.02(-0.350, 0.390) |
| P value | 0.989 | 0.921 | 0.914 |
